# Associations between cerebral blood flow and progression of white matter hyperintensities

**DOI:** 10.3389/fnimg.2024.1463311

**Published:** 2025-01-21

**Authors:** Siriluk Thammasart, Danielle J. Harvey, Pauline Maillard, Charles DeCarli, Corinne A. Donnay, Gregory J. Wheeler, Audrey P. Fan

**Affiliations:** ^1^Biological Engineering Program, Faculty of Engineering, King Mongkut's University of Technology Thonburi, Bangkok, Thailand; ^2^Public Health Sciences, University of California Davis School of Medicine, Davis, CA, United States; ^3^Neurology, University of California, Davis, Davis, CA, United States; ^4^Biomedical Engineering, University of California, Davis, Davis, CA, United States

**Keywords:** white matter hyperintensity (WMH), brain aging, penumbra, cerebral blood flow (CBF), arterial spin labeling (ASL), magnetic resonance imaging (MRI)

## Abstract

**Introduction:**

In an aging population, white matter hyperintensities (WMHs), observed on FLAIR MRI sequences, are indicators of cognitive decline, motor impairment, and increased vascular risk. However, the pathophysiological mechanisms underlying WMHs, including dynamic changes in cerebral blood flow (CBF) within and adjacent to lesions, remain poorly understood.

**Methods:**

Our study examined a diverse cohort of 300 elderly participants through arterial spin labeling (ASL) on 3 Tesla MRI, analyzing both cross-sectional and longitudinal data. We characterized the relationship between CBF and WMH development in different lesion locations (based on distance from ventricles) and brain tissue types (WMH lesion, penumbra, and normal white matter).

**Results:**

Our findings reveal that WMHs exhibit significantly lower relative CBF (rCBF) compared to penumbra, normal-appearing white matter, and gray matter, with juxtaventricular WMHs (JVWMH) displaying the most substantial reductions. Longitudinally, WMHs that increased in size over a two-year period had lower baseline rCBF than those that remained stagnant, particularly in juxtaventricular and periventricular regions.

**Discussion:**

This study not only highlights the predictive value of rCBF in WMH progression but also provides location-specific hemodynamic information about WMHs that can guide clinical management of WMH-related brain changes and their clinical manifestations.

## Introduction

White matter hyperintensities (WMHs) are a common imaging feature of aging brains that relate to cognition (Carmichael et al., 2010) and progress (e.g., increase in volume) over time (Maillard et al., 2011) WMHs are typically detected using fluid attenuated inversion recovery (FLAIR) MRI sequences and are considered a marker of white matter injury. These WMH lesions are known to increase with age and vascular risk factors (Nordahl et al., 2006) and negatively associate with gray matter volume (Wang et al., 2020). The volume of WMHs has been linked to an increased risk of cognitive (Benedictus et al., 2014) and motor (Silbert et al., 2008) impairment, stroke, dementia, as well as death (Debette and Markus, 2010). However, WMHs are also heterogeneous, with variable brain locations—juxtaventricular (JVWMH), periventricular (PVWMH), deep (DWMH), and juxtacortical lesions (Kim et al., 2008)—and different tissue properties (Jung et al., 2021; Shu et al., 2020) that underlie differential etiologies and associations with cognition. Furthermore, while WMHs are thought to reflect vascular injury to white matter, direct imaging evidence of ischemia within and surrounding lesions is limited, especially in longitudinal settings where causal inference is improved. As a result, characterizing perfusion status of WMHs and at-risk white matter tissue, and how it relates to the growth of WMH volume over time is critical to understand the pathophysiological nature of WMHs that may improve understanding of pathways to mitigate lesion progression and future clinical impairment.

WMH penumbra refers to the tissue surrounding WMHs that is more vulnerable than healthy white matter (WM) to convert to WMHs (Maillard et al., 2011). Thus, WMH penumbra may represent a clinically relevant target of reversible injury for interventions aimed at arresting the progression of WMHs (Wu et al., 2019). Previous studies showed decreased CBF and increased free water (FW) in tissue surrounding WMHs, suggestive of vascular and microstructural damage in the WMH penumbra region beyond the WMH core (Donnay et al., 2021). Longitudinal diffusion MRI studies have shown that changes in baseline fractional anisotropy (FA), FW (Mayer et al., 2022) and FLAIR signal intensities in a WMH penumbra defined within 8 mm of WMH lesions (Maillard et al., 2014) can predict enlarging WMHs at follow-up. FA values in the penumbral tissue of WMHs in the corpus callosum also contribute to cognitive deficits associated with subcortical ischemic vascular disease (Qiu et al., 2021), highlighting the importance of characterizing at-risk tissue to predict cognitive decline.

Prior studies show that WMHs are associated with overall cerebral perfusion deficits, although the findings are mixed. A recent meta-analysis suggested that the extent of WMHs may relate to lower whole-brain or gray matter (GM) perfusion (Benedictus et al., 2014; Crane et al., 2015). However, another study reported an association between higher WMH volume and reduced blood perfusion in WMHs, but not in normal-appearing white matter (NAWM) or GM (Van Dalen et al., 2016). Significantly lower CBF has been observed in the WMH penumbra area surrounding WMHs compared to the total brain NAWM, extending ~12 mm from both the established PVWMH and DWMH (Promjunyakul et al., 2015), with graded CBF decrease between lesions and NAWM observed in 473 elderly participants (Nasrallah et al., 2019). Low perfusion in NAWM has also been associated with subsequent WMH development (Promjunyakul et al., 2015). A few studies that directly compared diffusion (microstructural) vs. perfusion (physiological) deficits in WMH penumbra tissue have suggested that CBF changes are more diffuse than DTI changes around lesions (Wu et al., 2019; Promjunyakul et al., 2016). While existing imaging studies have linked WMHs to a general cerebral perfusion deficit, there remains a lack of comprehensive spatial and longitudinal characterization of perfusion status within WMHs. Although some studies have reported lower CBF in the penumbra area surrounding WMHs compared to total brain NAWM, there is a need for further investigation into the relationship between WMHs and perfusion deficits.

Therefore, this study aims to bridge these gaps by investigating CBF alterations in WMHs and their surrounding penumbra, across different WMH locations and growth patterns. We first conducted cross-sectional analysis of ASL for WMHs in various brain regions (juxta-, periventricular, and deep white matter) that are thought to reflect distinct pathogenic mechanisms and regional blood supplies. In follow-up scans, we assessed the relationship between WMH longitudinal category (stagnant, growing, or new WMH) and CBF values. Through this approach, we aim to identify white matter regions at risk of reduced blood flow and uncover potential perfusion biomarkers for predicting at-risk tissue in WMH progression, which may ultimately guide therapeutic interventions to prevent WMH accumulation.

## Methods

### Participants

The study included 208 individuals with normal cognition, 64 with mild cognitive impairment, and 28 with dementia, comprising a total cohort of 300 participants. All participants underwent standardized clinical evaluation at the University of California, Davis Alzheimer's Disease Research Center. Written informed consent was obtained from all participants, and the study received approval from the local ethics committee. Inclusion criteria were individuals age of 65 years of older, no contraindications to MRI, known cognitive status, and absence of other neurological conditions. Each participant underwent at least one brain MRI, with 90 participants undergoing a follow-up MRI ~2 years later. Table 1 provides a summary of participant characteristics at baseline.

**Table 1:** Summary of participant characteristics at baseline (*n* = 300).

**Variables**	**Mean (SD)**
Age (years)	77 (7.9)
Education (years)	14.7 (3.9)
Number of females	199
Percent with history of hypertension	68%
Mini-Mental State Examination (MMSE) score	28 (2.4)
Inter-scan interval (years); *n* = 90	1.9 (0.7)

### MRI processing

All brain imaging was performed at the University of California, Davis Imaging Research Center on a 3 Tesla Siemens Tim Trio system and 32-channel head receive coil. Structural scans included a 3-dimensional sagittal T1-weighted magnetization-prepared rapid gradient echo acquisition with voxel size of 1 mm^3^ isotropic, repetition time (TR) of 2,500 ms, echo time (TE) of 2.98 ms, and inversion time (TI) of 1,100 ms. A sagittal fluid attenuated inversion recovery (FLAIR) sequence was acquired with voxel size of 1.0 × 1.0 × 2.0 mm^3^, TR of 8,800 ms, TE of 496 ms, and TI of 2,360 ms. Perfusion measures were obtained using pulsed arterial spin labeling (ASL) with parameters including TR/TE: 4,000/13 ms, inversion time of 1,800 ms, bolus duration of 1,650 ms, voxel size of 4 × 4 × 10 mm^3^, 11 axial slices, and 60 control-label pairs for a total scan time of ~8 min (Luh et al., 1999). Perfusion-weighted difference signals were calculated by subtracting surrounding pairs before averaging. CBF maps were generated using a one-tissue compartment model and Bayesian Inference of Arterial Spin Labeling (BASIL) in the FMRIB software library (Chappell et al., 2008), assuming T1 relaxation in arterial blood of 1,650 ms, labeling efficiency of 0.98, and a blood-brain partition coefficient of 0.9 ml/g, according to consensus parameters (Alsop et al., 2015). WMHs were segmented from the FLAIR scans and T1-weighted images using a previously established, automated protocol based on image intensity histograms of different brain tissue types (DeCarli et al., 2005, 1992).

**Figure 1:**
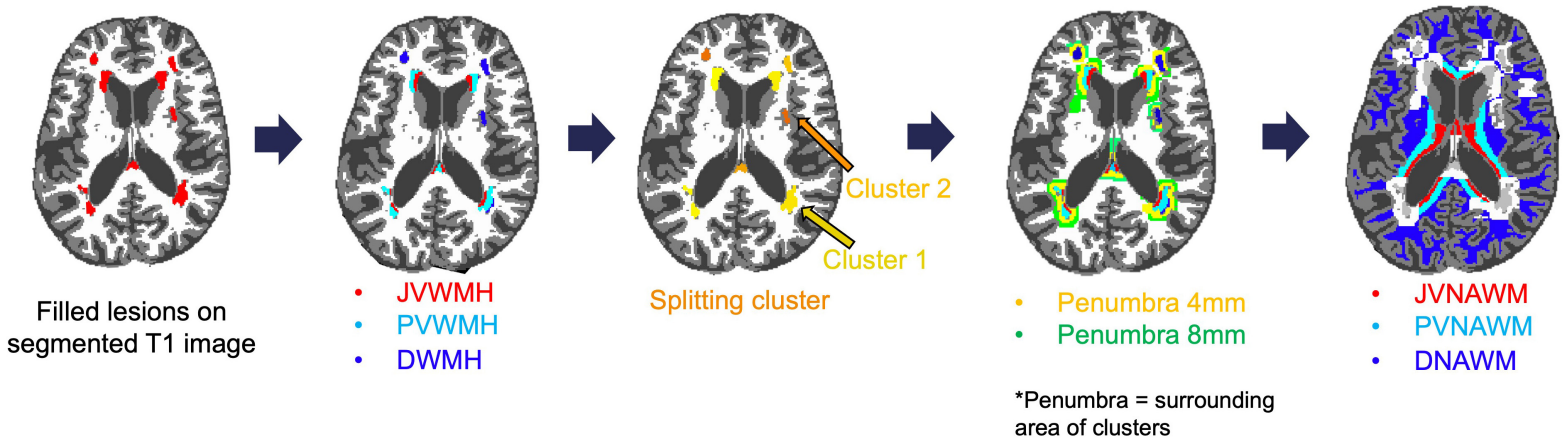
Example of creating a WMH map and splitting it into clusters (individual WMH lesions), penumbra, as well as location-specific mask for WMH and NAWM including juxtaventricular (JV), periventricular (PV), and deep (D) regions.

Tissue segmentation of T1-weighted images was performed to derive binary GM and WM masks using FSL (version 6.0.5.1), which included brain extraction and probabilistic tissue segmentation tools. Co-registrations of perfusion maps and binary masks of GM and WM to FLAIR images were conducted using FMRIB's Linear Image Registration Tool (FLIRT) (Jenkinson and Smith, 2001; Jenkinson et al., 2002). This step ensured that GM, WM, and WMH masks and CBF maps were in a common anatomical space for comparison across individuals. WMH masks were utilized to derive regional masks for JVWMH (within 3 mm from the ventricular surface) (Kim et al., 2008; Jung et al., 2021), PVWMH (between 3 and 10 mm) (DeCarli et al., 2005), and DWMH (10 mm or further) (Griffanti et al., 2018), as depicted in Figure 1.

Additionally, WMH FLAIR intensity penumbra, spanning ~3 to 8 mm around WMHs was identified in the previous studies (Maillard et al., 2011, 2014). Therefore, we assigned all WM voxels within 4- and 8-mm surrounding a WMH lesion to the 4- and 8-mm penumbra masks, respectively. NAWM masks were obtained by excluding WMH and WMH penumbra voxels from WM masks for each region.

The binary masks were then applied to the co-registered perfusion maps to compute CBF values for each brain tissue. The average CBF of GM, WMH, individual clusters, WMH penumbra, and NAWM, was quantified for each participant and normalized by dividing the values by the total brain CBF from the same individual.

### Longitudinal analysis and definition of growing, stagnant and new WMHs

For longitudinal evaluation (*N* = 90), we focused on lesion-specific analysis of mean baseline CBF in WMHs and WMH penumbras and how this associated with WMH progression. To evaluate WMH lesion development per participant, brain tissue segmentations were first linearly transformed between time points. At each time point, a WMH cluster (i.e., individual lesion) was defined as groups of voxels classified as WMHs in tissue segmentation, connected in a three-dimensional voxel neighborhood. Each WMH cluster in the follow-up image was visually inspected for overlap with a corresponding cluster in the baseline image, with lesions < 10 mm^3^ excluded from analysis (Gaubert et al., 2021).

WMH clusters were also split into categories based on their volume changes between baseline and follow-up (Supplementary Figure S1). Specifically, clusters were categorized as follows: (1) growing WMHs for lesions that show >10% volume increase; (2) stagnant WMHs for baseline lesions that show < 10% volume change; and (3) new WMHs for those lesions found only in the follow-up scans. For each WMH cluster, all WM voxels within 4- and 8-mm surrounding the lesion were assigned separate WMH penumbra regions of interest (ROIs). Each WMH penumbra ROI was also classified as “stagnant,” “growing,” or “new” based on the longitudinal nature of its corresponding WMH. We calculated CBF within individual WMH clusters and their penumbras for longitudinal analysis, and all perfusion values were again normalized to the participant's total brain CBF.

### Statistical analysis

Statistical analysis was performed using R version 4.2.2 (R Foundation) with a significance level of 0.05. Prior to regression, CBF values and WMH volumes were normalized to total brain CBF and volume, respectively, yielding relative CBF (rCBF) and relative volume (rVol). Both rCBF and rVol values were log-transformed before use in mixed-effects models to address non-normality of distributions.

#### Cross-sectional analysis

We conducted cross-sectional analyses comparing rCBF across gray matter (GM), white matter (WM), WMH, and WMH penumbral regions in all 300 participants. The penumbral regions were defined as 4 mm (P4) and 8 mm (P8) around the WMH boundaries, as well as normal-appearing white matter (NAWM). To assess the impact of tissue type and brain location on rCBF, we used a linear mixed-effects model. Tissue types (WMH, P4, P8, NAWM) and brain locations [juxtaventricular (JV), periventricular (PV), and deep (D)] were included as fixed effects. Random intercepts for individual participants were included to account for within-subject variability and repeated measures. Two participants were excluded due to a lack of WMHs in the PV or D regions.

#### Association between rCBF and WMH volume

A separate mixed-effects model was used to explore the association between rCBF and rVol across brain locations (JV, PV, and D), incorporating an interaction term between rVol and location. To aid interpretation, the log-transformed rVol values were mean-centered by subtracting the average log-transformed WMH volume across all participants. This allowed us to interpret the main effect of location on rCBF at a lesion size of 125 mL.

#### Longitudinal analysis

For the longitudinal analysis, lesion-specific models were fit with rCBF values for each WMH cluster as the dependent variable. In these models, the longitudinal category (stagnant, growing, and new) of the lesion, the session (baseline vs. follow-up), and their interaction were included as fixed effects. Independent lesion-specific models were created for each location (juxtaventricular, periventricular, deep), which also included person-specific random intercepts. Similar mixed-effects regression models were fit for longitudinal WMH penumbra rCBF values.

## Results

### Cerebral blood flow in GM, NAWM, WMH, and WMH penumbra

**Figure 2:**
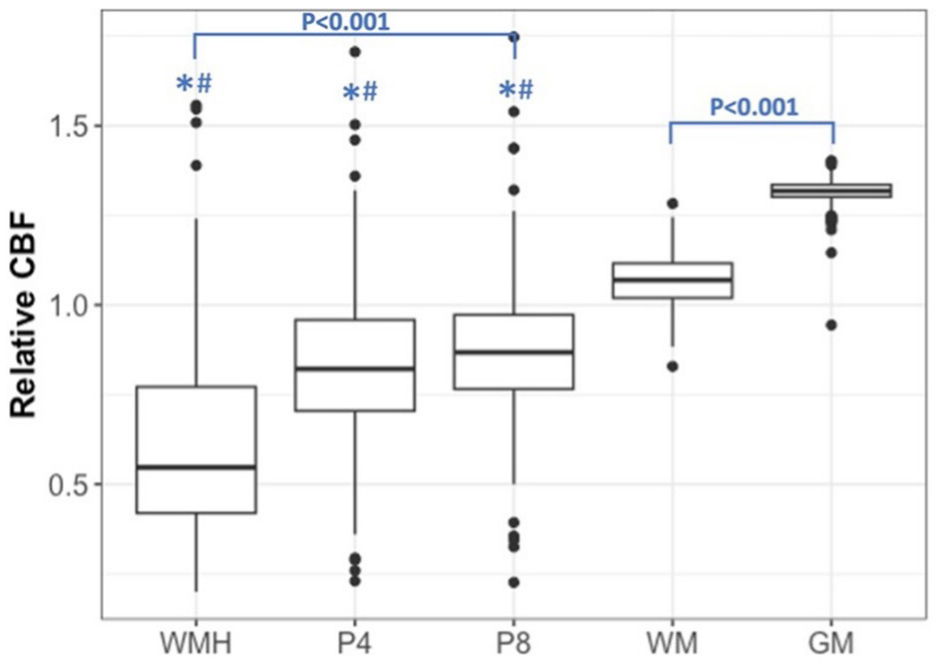
Relative cerebral blood flow in the gray matter (GM), white matter (WM), white matter hyperintensities (WMH), WMH penumbra-4 mm (P4), and WMH penumbra-8 mm (P8). Boxplots of rCBF for 300 participants illustrate the distribution of rCBF across different tissue types. *P < 0.001 vs. GM. ^#^*P* < 0.001 vs. WM.

Cross-sectional comparisons of rCBF across brain tissues in 300 individuals, as shown in Figure 2, highlight lower rCBF within white matter hyperintensities (WMH) compared to all other tissues. Specifically, the mean total brain-adjusted rCBF for WMHs (Mean ± Standard error 0.814 ± 0.015) and the penumbral regions at 4 mm (0.845 ± 0.008) and 8 mm (0.880 ± 0.007) were significantly reduced compared to the mean rCBF of normal-appearing white matter (WM, 1.067 ± 0.004) and gray matter (GM, 1.315 ± 0.002). The rCBF within WMHs was also found to be significantly lower than that within the 8 mm WMH penumbral areas (*P* < 0.001), but not the 4 mm WMH penumbra area.

### Differential CBF in WMH, penumbra, and NAWM across white matter locations

**Figure 3:**
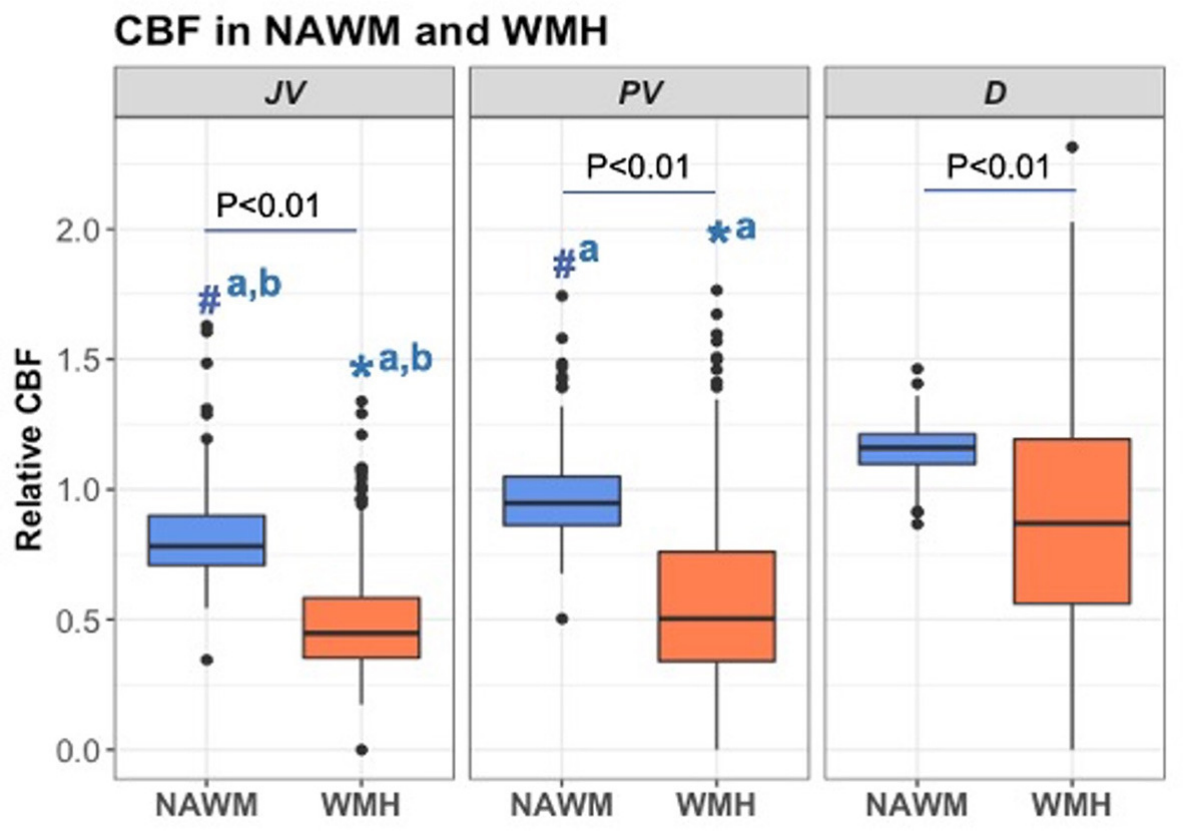
Relative cerebral blood flow in the normal-appearing white matter (NAWM) and white matter hyperintensities (WMH) across juxtaventricular (JV), periventricular (PV), and deep (D) areas. All data were log-transformed to normalize population variance and compared using mixed-effect models. Boxplots of rCBF for 298 participants by tissue type and location are presented. #^a^*P* < 0.001 vs. DNAWM. #^a, b^*P* < 0.01 vs. PVNAWM and DNAWM. *^a^*P* < 0.001 vs. DWMH. *^a, b^*P* < 0.001 vs. PVWMH and DWMH.

Comparison of mean rCBF between NAWM and WMH across distinct brain locations (Figure 3) consistently demonstrated lower CBF in WMH relative to NAWM within each respective location. Notably, JVWMH showed significantly lower rCBF than PVWMH (Mean ± SEM: 0.495 ± 0.012 vs. 0.592 ± 0.020, *P* < 0.001) and DWMH (Mean ± SEM: 0.892 ± 0.024, *P* < 0.001). Similar patterns were observed in NAWM, with JVNAWM demonstrating significantly lower rCBF compared to PVNAWM (Mean ± SEM: 0.816 ± 0.009 vs. 0.973 ± 0.009, *P* < 0.001) and DNAWM (Mean ± SEM: 1.156 ± 0.005, *P* < 0.001). Furthermore, deep regions exhibited significantly higher rCBF than JV regions (beta = 0.123, *P* < 0.001), while no significant difference in rCBF was found between PV and JV locations (beta = 0.012, *P* = 0.246). These observations were consistent with a linear mixed-effects model that evaluated rCBF in lesions with distance from the WMH centroid to the ventricle as a covariate. This model showed that lesions close to ventricles had lower CBF values compared to lesions further away from the ventricles (Supplementary Figure S2).

### Association between WMH volume and WMH CBF across locations

**Figure 4:**
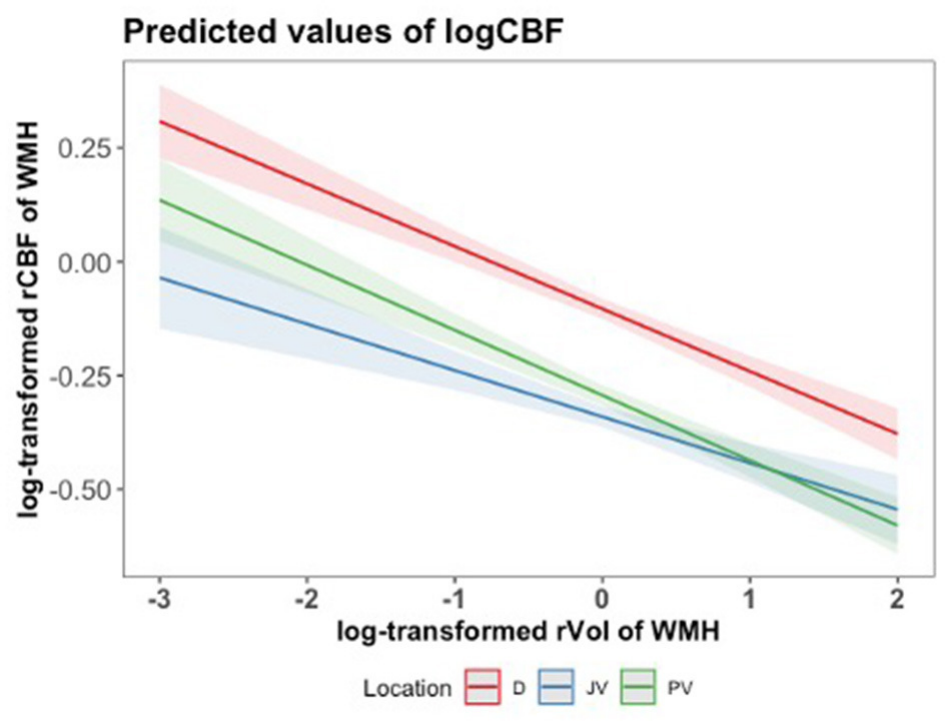
Predicted values of log-transformed CBF (logCBF) as a function of log-transformed relative volume of WMH (log rVol) across brain locations (JV, PV, D), with mean-centered log rVol values. Lines represent the estimated regression line with a 95% confidence interval, based on the mixed-effects regression model. A log-transformed rVol of WMH equal to 0, for instance, relates to 125 mL.

In our analysis, the log-transformed cerebral blood flow (logCBF) exhibited an inverse relationship with the log-transformed relative volume of white matter hyperintensities (rVol of WMH) across different brain locations (Figure 4). We found a significant inverse relationship between log-transformed rCBF and log-transformed relative volume of WMHs (rVol of WMH) across all brain locations (Figure 4). We consistently observed that larger WMH volumes were associated with lower CBF, with the PV region demonstrating the steepest decline in logCBF with WMH volume. This finding was supported by linear mixed-effects model results, showing significant interactions between brain volume and location in predicting cerebral blood flow (PV: beta = −0.1897, *t* = −15.406, *p* < 0.001; JV: beta = −0.2369, *t* = −19.240, *p* < 0.001). Individuals with lesions of average size (logVol = 0) had higher rCBF in deep WMHs than in other locations, which is consistent with Figure 3. Although rCBF was lower in regions with larger WMH volumes, the mean WMH volume did not differ significantly across the brain locations, as determined by an ANOVA test (Supplementary Figure S3).

### Lesion-specific baseline rCBF and longitudinal WMH progression

Given the observed regional disparities in regional cerebral blood flow (rCBF), we analyzed lesion-specific longitudinal changes in perfusion separately within JV, PV, and deep brain regions, as detailed in Table 2. For each location, we investigated whether baseline rCBF differed between stagnant, growing, and new white matter hyperintensities (WMH) categories. Our findings consistently showed that growing WMHs exhibited significantly reduced baseline rCBF compared to their stagnant counterparts in both JV and PV areas, with *P*-values ranging from 0.018 to 0.021. This reduction in rCBF was also noted within the 4- and 8-mm penumbral zones surrounding growing WMHs in the JV and PV regions, emphasizing the potential of baseline rCBF as a biomarker for predicting WMH progression. Interestingly, in the deep brain regions, growing WMHs demonstrated an increase in baseline rCBF relative to stagnant WMHs, as indicated by a positive mean difference, suggesting a divergent pathophysiological mechanism at play in different WMH locations.

**Table 2:** Differences in baseline rCBF in growing and new WMH compared to stagnant WMHs and penumbra across locations.

**Tissue**	**Stagnant**	**Category: growing WMHs**			**Category: new WMHs**		
	**(Intercept)**	**Mean difference**	**95% CI**	***P*** **-value (corrected)**	**Mean difference**	**95% CI**	***P*** **-value (corrected)**
JVWMH	−0.295	−0.127	(−0.209, −0.045)	0.018^*^	−0.105	(−0.222, 0.011)	0.119
JVP4	−0.234	−0.100	(−0.168, −0.033)	0.018^*^	−0.088	(−0.186, 0.009)	0.119
JVP8	−0.109	−0.070	(−0.125, −0.015)	0.021^*^	−0.067	(−0.149, 0.015)	0.144
PVWMH	−0.294	−0.112	(−0.197, −0.028)	0.021^*^	−0.213	(−0.352, −0.074)	0.018^*^
PVP4	−0.225	−0.095	(−0.164, −0.027)	0.021^*^	−0.164	(−0.277, −0.052)	0.018^*^
PVP8	−0.176	−0.071	(−0.127, −0.015)	0.021^*^	−0.111	(−0.205, −0.018)	0.060
DWMH	−0.343	0.098	(0.011, 0.184)	0.035^*^	0.113	(−0.014, 0.239)	0.119
DP4	−0.23	0.062	(−0.001, 0.125)	0.062	0.054	(−0.041, 0.149)	0.287
DP8	−0.154	0.037	(−0.014, 0.089)	0.158	0.037	(−0.039, 0.112)	0.334

Values are mean differences (95% CI) in log-transformed rCBF (logCBF) between new or growing WMH categories compared to the stagnant WMH category, analyzed separately by tissue location and repeated for WMH penumbra rCBF values. The initial *p*-values are derived from lesion-specific mixed-effects regression models. Adjusted *p*-values reflect corrections for multiple comparisons using the False Discovery Rate (FDR) method to control the expected proportion of type I errors (false discoveries) across all tests. Asterisks indicate *p*-values that are considered significant after correction.

New PVWMHs also showed lower baseline CBF in the tissue regions of interest that eventually became WMH lesions and P4 penumbra at follow-up. Conversely, in new JVWMHs, similar trends of reduced rCBF were observed, although these did not reach statistical significance, suggesting that the location influences the relationship between initial perfusion status and new WMH development.

## Discussion

This study aimed to investigate blood flow patterns surrounding WMHs in elderly individuals across various cognitive abilities. Three key findings emerged. First, on cross-sectional analyses, WMHs and their surrounding penumbra exhibited notably lower perfusion compared to both the total brain's gray matter and adjacent NAWM. Second, there was pronounced regional variation in relative CBF (rCBF) across white matter areas, with deep white matter regions showing higher perfusion than their juxtaventricular and periventricular counterparts. Thirdly, notable heterogeneity in rCBF was observed across various longitudinal WMH trajectories, defining between stagnant, growing, and newly formed lesions. Specifically, compared to stagnant lesions, baseline rCBF was lower in growing and new WMHs, indicating a likely cause for their expansion or development.

Further, our cross-sectional analysis found that WMHs are associated with reduced rCBF compared to NAWM and gray matter. The observed decrease in rCBF within WMHs supports the notion that these lesions are linked to impaired perfusion and altered cerebrovascular dynamics. Furthermore, the areas surrounding WMHs, particularly the 4-mm and 8-mm WMH regions, exhibited distinct rCBF patterns and demonstrated lower rCBF compared to the brain's normal-appearing white matter. This observation emphasizes the potential susceptibility of the surrounding tissue to ischemic insults and supports the notion of compromised vascular health in the vicinity of WMHs (O'Sullivan et al., 2002).

The functional, histopathological, and etiological characteristics of WMHs are influenced by their white matter vascular supply, which depends on location relative to the lateral ventricles (Kim et al., 2008). The PV region, supplied by arteriole terminals of medullary circulation with diameters < 100 μm (Moody et al., 1990), appears more susceptible to large-vessel disease (DeReuck, 1971) and ischemic hypoperfusion (Akashi et al., 2017). Juxtaventricular areas, lying within 3-mm (Kim et al., 2021) of the ventricular surface and directly adjacent to it, may also contain non-ischemic WMHs that may result from cerebrospinal fluid (CSF) leakage (Khan et al., 2020). While WMHs close to the ventricles are more likely influenced by hemodynamic factors, DWMHs in contrast are associated with small vessel disease. It has been observed that DWMHs, but not PVWMHs, are specifically linked to vascular dementia (Smith et al., 2016). Moreover, differences in small-vessel circulation within the white matter between deep and PV regions exist. This microvascular architecture aligns with the finding that lower cortical CBF is associated with a greater burden of PVWMH (Bahrani et al., 2017), emphasizing the importance of region-specific analysis in WMHs.

Our study showed that both JV and PV regions displayed a statistically significant reduction in rCBF within WMHs compared to NAWM (*P* < 0.01), as depicted in Figure 3. Similarly, deep white matter regions demonstrated a notable decrease in CBF within WMHs relative to NAWM (*P* < 0.01). These findings were consistent across all examined regions, suggesting a pervasive pattern of reduced perfusion in areas affected by WMH. In lesions, this finding may reflect additional perfusion reduction on top of the normal rCBF variations in different NAWM locations, i.e., rCBF was also greater in deep vs. JV/PV areas for NAWM. In NAWM, regional rCBF differences may reflect delays in arterial transit time that varies across white matter areas and are accentuated by underlying pathology. The fact that rCBF was lower in periventricular compared to deep white matter is consistent with a previous ASL study that showed lower periventricular CBF compared to the rest of the white matter in a large, middle-aged cohort (Dolui et al., 2019).

There was an inverse relationship between WMH volume and rCBF across all locations, with WMH volume negatively affecting rCBF in JV and PV regions more severely than in deep regions in Figure 4. Our findings align with a meta-analysis of 34 studies (Stewart et al., 2021) that demonstrated an association between larger WMH volumes and lower CBF across different locations. However, in another study of community-dwelling elderly participants, WMH burden was not directly associated with cortical perfusion at baseline after adjusting for the effect of age (Han et al., 2022). Our results reinforce that the extent of WMH volume plays a significant role in CBF reduction specifically in the lesion, which varied with the location of the WMH. While the volume of WMHs did not show significant differences across regions, the interaction effects in our model highlight that its regional impact on rCBF varied across locations, which may have different vascular health or disease severity.

The strengths of our study lie in its longitudinal design to investigate rCBF with the progression of WMHs over time. The use of the same scanner and imaging protocol at both baseline and follow-up ensured consistency and minimized potential confounders in differentiating between growing and new WMHs based on FLAIR intensity. Specifically, segmenting voxels at the interface between these tissues can introduce a partial volume effect, which may impact the accuracy of the analysis (De Groot et al., 2013). We focused on lesions with volume of at least 10 mm^3^ to avoid sporadic lesions reflective of image noise. Our definition of stagnant, new, and growing is consistent with Maillard et al. (2014), and consistent criteria are important in future work for comparisons across studies.

In our longitudinal analysis, we observed a significant reduction in baseline rCBF in growing WMHs compared to their stagnant counterparts within JV and PV regions, aligning with previous reports that have associated impaired perfusion with WMH expansion (Zhang et al., 2022). Growing WMHs also had lower baseline mean CBF within their penumbras when compared to stagnant WMHs, especially for JV and PV areas and for the 4- and 8-mm WMH penumbra zones. This finding suggests that baseline CBF measurements have the potential to identify WMH penumbra areas at a higher risk of transitioning into growing or new WMHs, distinguishing them from areas that are likely to remain unchanged during follow-up. This observation is consistent with previous studies showing that baseline CBF can predict development of new WMH over an 18-month follow-up interval (Promjunyakul et al., 2015). Similar to expanding lesions, new WMHs were also associated with lower baseline rCBF specifically in the PV area (Promjunyakul et al., 2018). The PV region is known to have unique vascular architecture, lying in the watershed region that includes terminal zones of penetrating vessels (Abramowicz, 1964). This anatomy might render this area more vulnerable to hypoperfusion or microvascular changes that contribute to the observed reduction in CBF within WMHs (Lin et al., 2017). Alternatively, periventricular WMHs result from venous collagenosis, whereas deep WMHs are associated with diminished blood flow with hypoxia (Moody et al., 1995).

The absence of this longitudinal rCBF pattern in expanding or new deep WMHs or their penumbras suggests different pathophysiology of deep WMHs compared to other locations. Another study reported an association of both CBF and structural markers within the WMH penumbra layers 1–5 with PWMH growth during follow-up, but CBF alone was not independently predictive of DWMH expansion (Promjunyakul et al., 2018). The divergent WMH subtypes likely reflect anatomical and pathological variations: JV regions are affected mainly by cerebrospinal fluid leakage, PV regions are susceptible to chronic insufficiency and watershed infarctions due to their location in a cerebral perfusion watershed area (Thomas et al., 2003), while deep WMHs are often associated with arteriolosclerosis, leads to localized ischemic areas (Lustig et al., 2009). Growing deep WMHs may also display compensatory mechanisms that lead to increased baseline rCBF compared to stagnant deep WMHs. These findings emphasize the importance of anatomical location of WMHs when evaluating their effects on CBF and the distinctive influence of periventricular regions in the context of WMH-related hemodynamic changes over time.

Our study has several limitations to consider. Firstly, the small sample size during the second visit may limit the generalizability of our findings. Secondly, the exclusion of small lesions (< 10 mm^3^) may impact detection in a longitudinal study, especially in new lesions that tend to be smaller. The classification of stagnant vs. growing lesion classification based on a 10% volume change threshold between baseline and follow-up was empirically chosen and warrants further optimization in future studies. Thirdly, our relatively short follow-up period limits longer-term understanding of WMH progression and should be extended in future studies.

The ASL MRI method we adopted for perfusion assessment also warrants several technical considerations. Due to the small blood volume and low perfusion signal in WM voxels, large voxels were adopted for ASL scans which may have led to some partial volume bias. Currently, partial volume correction methods are underutilized and typically perform gray-white matter segmentation on brain structural images to correct the concomitant CBF maps (Zhao et al., 2017). Although this processing step is a keen area of interest for future perfusion analysis in studies of older populations where atrophy is expected, a recent review found that experimental results to date have not shown a consistent benefit of correction for PVE (Chappell et al., 2021). This lack of consensus may reflect fewer systematic studies that perform partial volume correction and variable definitions of gray-white matter thresholds. A future direction of our ASL study is take advantage of ongoing methodological advances to achieve partial volume correction on CBF maps, which are recommended to be reported alongside conventional analyses as in this manuscript. We also note that Figure 4 shows an inverse relationship between rCBF and rVol of white matter lesions, including in hyperintensities close to the ventricles that are most likely to experience partial volume effects with cerebrospinal fluid. After partial volume correction, smaller PV and JV lesions are likely to have higher rCBF (i.e., less contribution from CSF signal, which has low perfusion signal); therefore, the trend in Figure 4 is expected to be stronger after partial volume correction.

To improve signal-to-noise ratio of the perfusion measures, alternative labeling strategies such as pseudo-continuous ASL sequences should be considered. Our primary metric of interest was a relative CBF measure to account for normal physiological variations across individuals in total CBF. Future work may investigate the value of absolute CBF values, which requires correction of CBF values for longer arterial transit times (ATT) in WM. If multiple post-label delays are used with ASL MRI, improved CBF quantification may be achieved, alongside measures of ATT, which independently associate with WMH (Zhang et al., 2022). Oxygen extraction fraction from quantitative BOLD (blood oxygenation level dependent) MRI is an additional marker of brain vascular health that is perturbed in ischemic conditions and will provide further insight into the etiology of WMHs (Le et al., 2023). Holistic imaging of these MRI physiological measures in future work will contribute to a better understanding of WMHs and their impact on cerebral perfusion, especially in the context of cognitive status, which may be mediated by these confounders.

## Conclusion

Our study underscores the importance of considering lesion location and baseline perfusion status when evaluating WMH progression in elderly populations with and without cognitive impairment. Baseline CBF measurements within WMHs and their penumbras, particularly in growing juxtaventricular and periventricular areas, have the potential to predict WMH progression and identify at-risk tissue. Our findings hold significant clinical implications, suggesting that arterial spin labeling MRI could be instrumental in identifying individuals at higher risk for WMH progression, thereby informing targeted preventive and therapeutic strategies.

## Data Availability

Data for this article and Supplementary material is available upon request directed to the corresponding author.
