# Supplementary material for: Associations between cerebral blood flow and progression of white matter hyperintensities
Source: Front Neuroimaging. 2025 Jan 21;3:1463311. doi: 10.3389/fnimg.2024.1463311 (PMC11790564; doi:10.3389/fnimg.2024.1463311)
Supplement: Supplementary file 1 [file Table_1.docx]

Supplementary Material

# Supplementary Figures


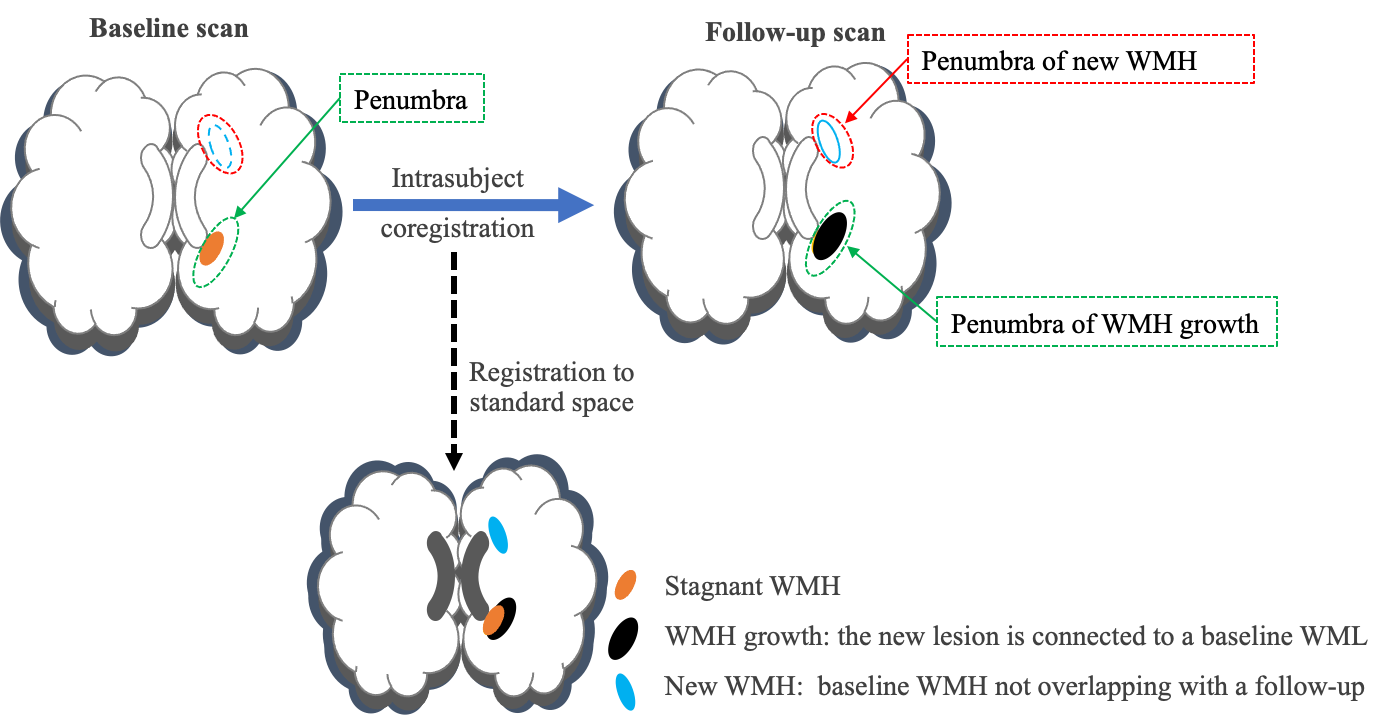


**Figure S1.** A schematic overview of the spatial normalization procedure for two scans of the same participant. The baseline scan is linearly registered to the follow-up scan, creating an intermediate image. Then, both images are non-linearly registered to the 1mm isotropic MNI template where lesions are categorized as WMH stagnant, WMH growth or new WMH. The stagnant WMH and WMH growth, which appear in the follow-up images, are represented in solid orange and black, respectively, and are overlaid onto the baseline image. The boundary of new WMHs in the baseline image is indicated by a blue dashed line, which has been transferred from a cluster mask of the follow-up image (solid blue) to the baseline image. The boundaries of the penumbras of growing and stagnant baseline clusters are depicted with green dashed lines on the baseline image. The boundaries of the penumbras of new clusters are depicted with red dashed lines on the follow-up image and are superimposed on the baseline image.

**
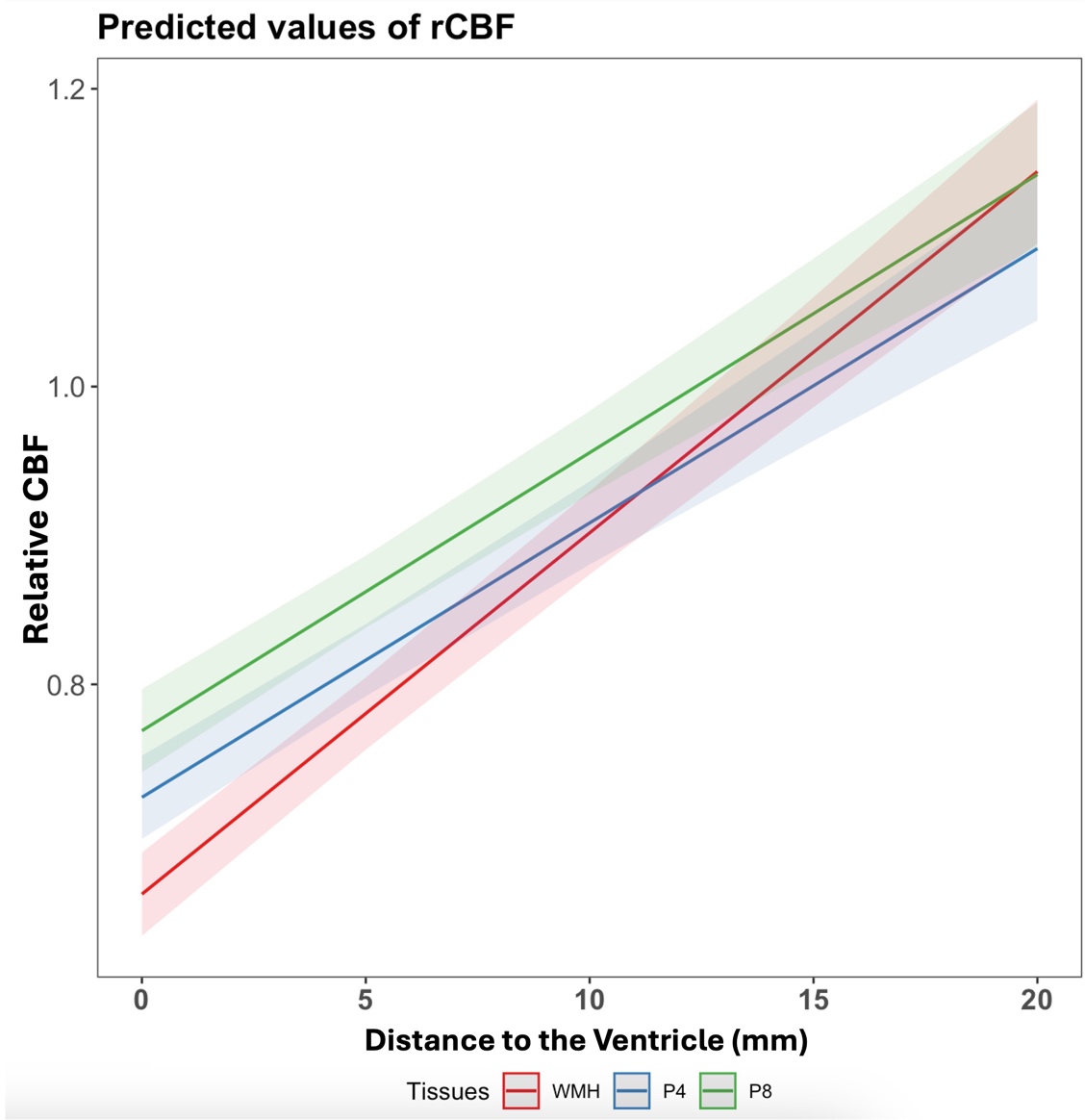
**

**Figure S2.** Predicted relative cerebral blood flow (rCBF) displayed against distance from the WMH lesion centroid to the ventricle. Regression lines are from mixed-effects modeling and are shown by tissue type (WMH, P4, P8). Shaded areas represent the 95% confidence intervals.

**
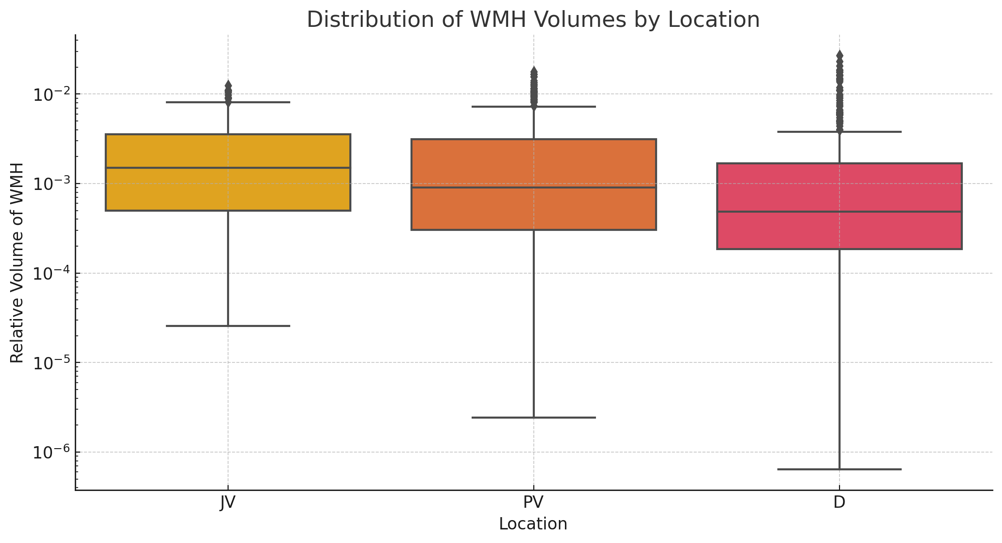
**

**Figure S3.** White matter hyperintensities (WMH) volumes by location for juxtaventricular (JV), periventricular (PV), and deep (D) areas. ANOVA test results comparing the mean WMH volumes across the three locations. The p-value of 0.397 indicates no statistically significant difference in the WMH volumes between the locations.
